# Supplementary material for: Endogenous cannabinoids in the piriform cortex tune olfactory perception
Source: Nat Commun. 2024 Feb 9;15:1230. doi: 10.1038/s41467-024-45161-x (PMC10858223; doi:10.1038/s41467-024-45161-x)
Supplement: Supplementary file 1 — Supplementary Information [file 41467_2024_45161_MOESM1_ESM.pdf]

Endogenous cannabinoids in the piriform cortex tune olfactory perception

Supplementary Information

Content:

- Supplementary Figures 1 to 11
- Supplementary Tables 1 and 2
- Supplementary References

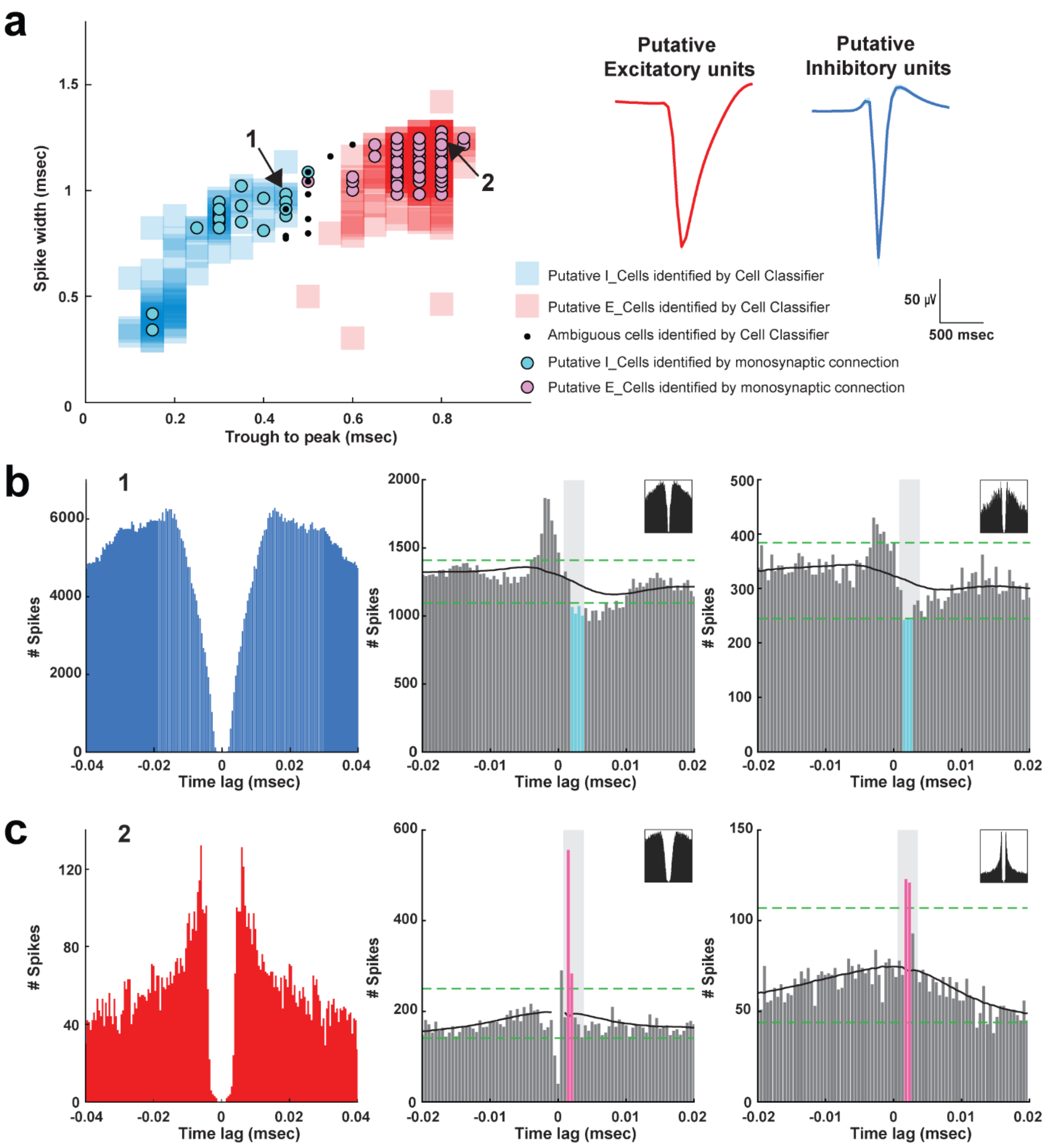

Supplementary Figure 1. Cell-type classification in the anterior piriform cortex.

(**a**) Left: distribution of the spike waveform features of putative excitatory (E_Cells, red), inhibitory (I_Cells, blue) and ambiguous units (black) identified by a cell classifier[1] (square) and by putative monosynaptic connections[2] (script adapted from ref.[3]) (circle) for experiments in freely moving

mice. Right: averaged waveforms across all putative excitatory and inhibitory units classified with the cell classifier. Mean, bold line; SEM, shaded area; n = 717 and n = 102 from 9 mice for excitatory and inhibitory units, respectively. (**b**) Example of I_Cell [from arrow 1 in (**a**)] and (**c**) example of E_Cell [from arrow 2 in (**a**)] illustrating putative monosynaptic connections. Left, auto-correlograms for reference units. Middle-right, cross-correlograms between reference and two target units (black line: predicted values; green dotted lines: upper and lower significance thresholds with p = 0.001, insert: auto-correlograms for the target units). Significant bins in the monosynaptic time window (1 to 4 msec) are plotted in light blue (inhibitory connection) and pink (excitatory connection).

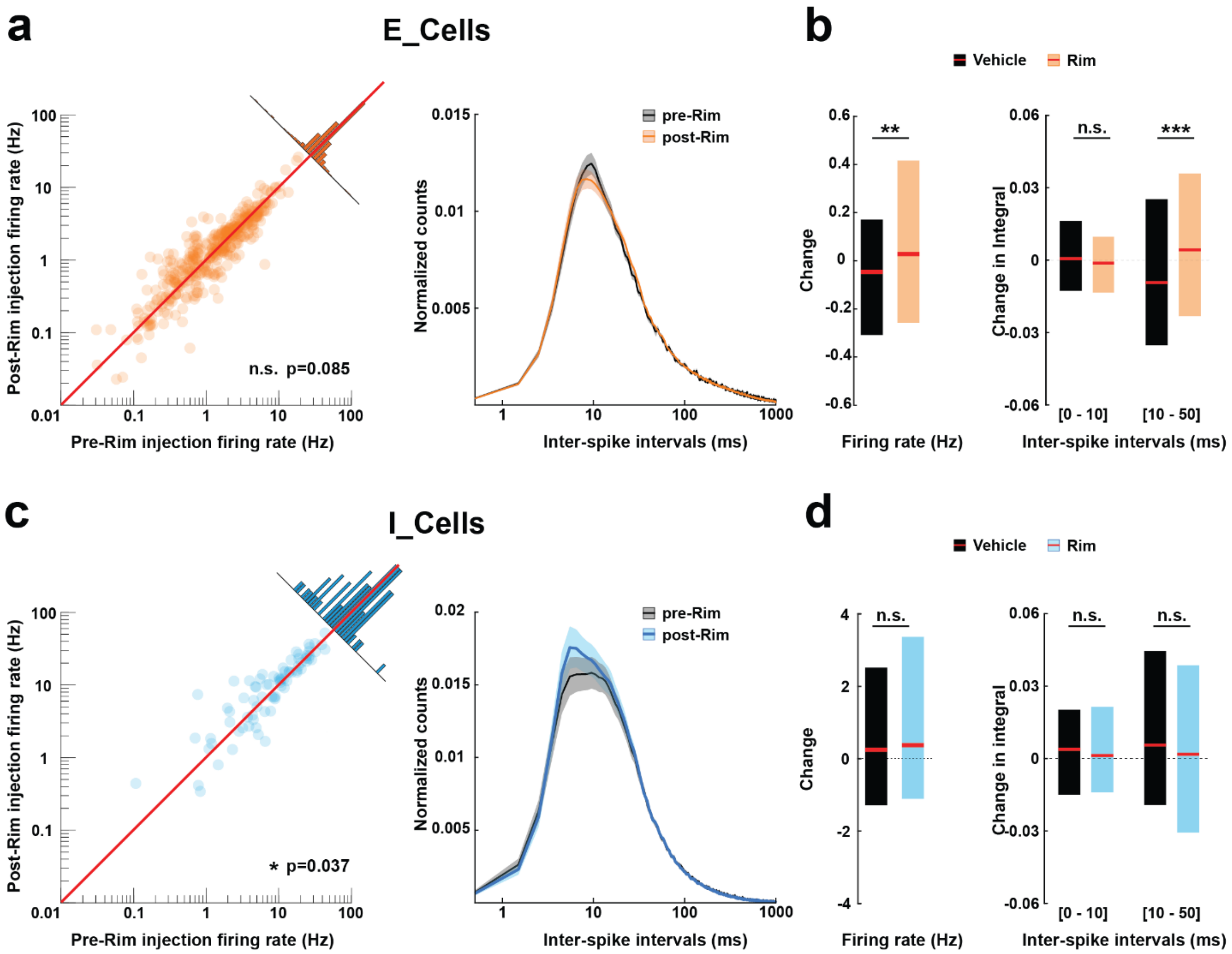

Supplementary Figure 2. Effect of CB1 receptor blockade on aPC firing pattern.

(**a**) Left: distribution of the firing rates for putative excitatory units (E_Cells) before (Pre) and after (Post) injection of CB1 receptor antagonist Rimonabant (Rim). Red line, no change. Pre: 1.41 [0.58 2.79] Hz *vs* Post: 1.46 [0.63 2.82] Hz. Two-sided Wilcoxon's paired signed rank test: P = 0.085. n = 393 E_Cells from 11 sessions (8 mice). Right: distribution of the interspike-intervals Pre (black) and Post (orange) injection of Rim. Mean, bold line; SEM, shaded area. n = 305 E_Cells from 11 sessions (8 mice). (**b**) Left: median change in firing rates for E_Cells. Vehicle: -0.05 [-0.31 0.17] Hz *vs* Rim: 0.03 [-0.26 0.42] Hz. Two-sided Mann-Whitney test: **P = 0.002. n = 645 and n = 393 E_Cells from 17 sessions (9 mice) and 11 sessions (8 mice) for Vehicle and Rim, respectively. Right: median change in integral intervals from the interspike-interval curves of E_Cells. Vehicle *vs* Rim ($x10^{-3}$): from 0 to 10 msec, 0.62 [-12.67 16.19] *vs* -0.79 [-13.39 10.51]; from 10 to 50 msec, -9.27 [-35.14 25.13] *vs* 4.23 [-23.11 35.80]. Two-sided Mann-Whitney test: P = 0.107; ***P = 0.003; for the 2 intervals; n = 456 and n = 305 E_Cells from 17 sessions (9 mice) and 11 sessions (8 mice) for Vehicle and Rim, respectively. (**c**) Left: distribution of the firing rates for I_Cells before (Pre) and after (Post) injection of Rim. Pre: 8.59 [3.88 17.55] Hz *vs* Post: 11.05 [4.30 18.17] Hz. Two-sided Wilcoxon's paired signed rank test: *P = 0.037. n = 78 I_Cells from 9

sessions (6 mice). Right: distribution of the interspike-intervals pre (black) and post (blue) injection of Rim. Mean, bold line; SEM, shaded area. n = 77 I_Cells from 9 sessions (6 mice). (**d**) Left: median change in firing rates for putative inhibitory units (I_Cells). Vehicle: 0.23 [-1.30 2.50] Hz *vs* Rim: 0.36 [-1.12 3.35] Hz. Two-sided Mann-Whitney test: P = 0.69. n = 80 and n = 78 I_Cells from 13 sessions (7 mice) and 9 sessions (6 mice) for Vehicle and Rim, respectively. Right: median change in integral intervals from the interspike-interval curves of I_Cells. Vehicle *vs* Rim ($x10^{-3}$): from 0 to 10 msec, 3.78 [-14.98 20.16] *vs* 1.25 [-13.99 21.38]; from 10 to 50 msec, 5.55 [-19.13 44.38] *vs* 1.74 [-30.55 38.50]. Two-sided Mann-Whitney test: P = 0.823; P = 0.758; for the 2 intervals, respectively; n = 79 and n = 77 I_Cells from 13 sessions (7 mice) and 9 sessions (6 mice) for Vehicle and Rim, respectively. Boxplots and values in legend represent median and 25-75th percentiles ([lower bound upper bound]). n.s., non-significant. Source data and additional distribution characteristics are provided in the **Source Data** file.

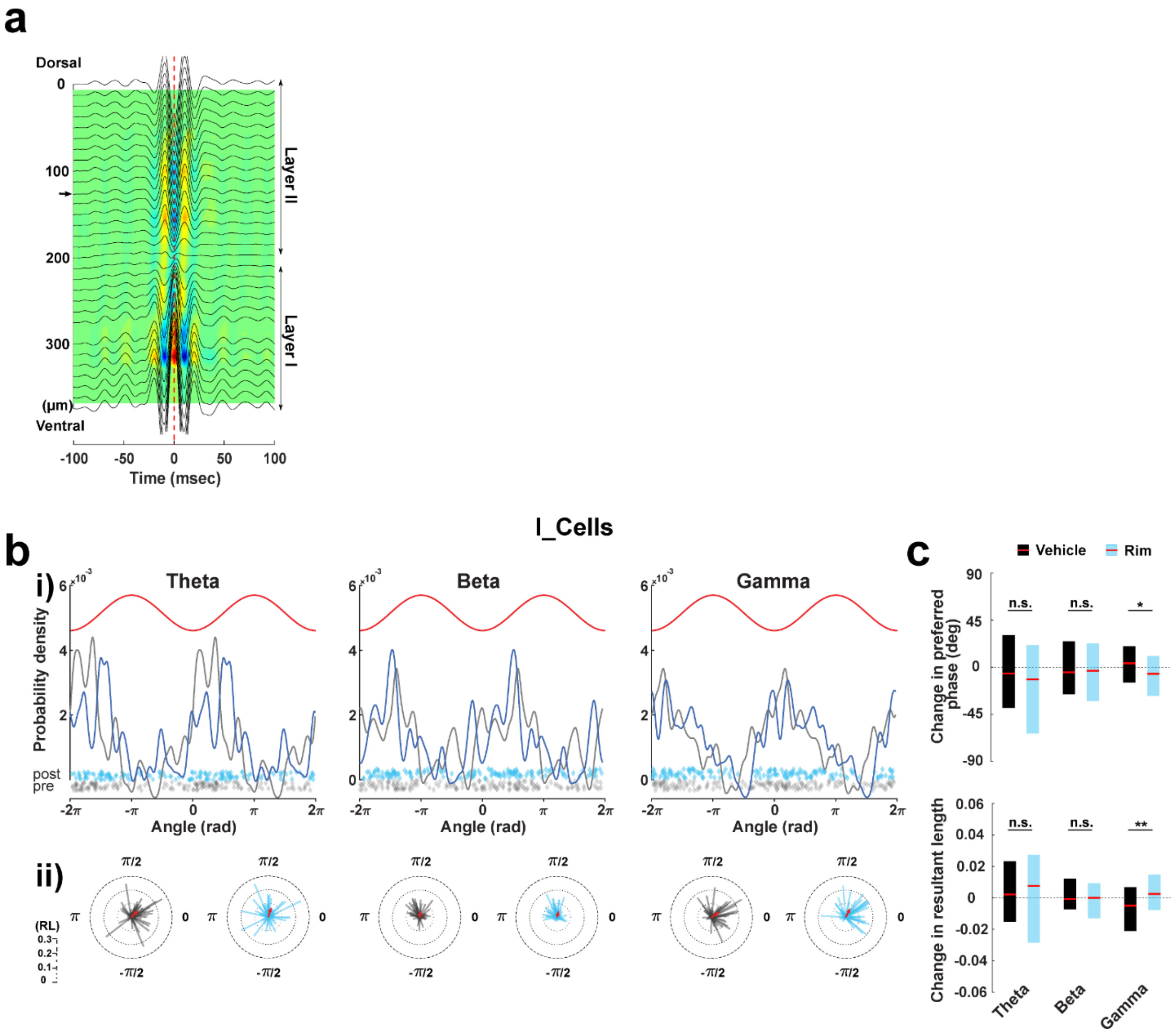

Supplementary Figure 3. Gamma oscillations in the anterior piriform cortex and effect of CB1 receptor blockade.

(**a**) Example of current source density (CSD) aligned on gamma epochs in the anterior piriform cortex (aPC) (zero corresponds to gamma trough on the reference channel [black arrow]; see **Methods**). Black superimposed traces represent the averaged LFP signals recorded on 30 channels of the silicon probe (two non-functional channels were excluded). (**b**) Same as (**Fig. 2d**) but for putative inhibitory units (I_Cells), pre- (grey) or post- (blue) systemic injection of Rimonabant (Rim). **i)** Top: red: theoretical cycles. Curves: probability densities of preferred phase. Bottom (diamonds): preferred phase of individual units. **ii)** Polar distributions of the preferred phases and resultant lengths (RL) for individual units, pre- and post-Rim injections. Red vectors: mean preferred phases and RL for the population. n = 77 units from 9 sessions (6 mice). (**c**) Same as (**Fig. 2e**) but for I_Cells. Top: Median change in preferred phase. Theta: Vehicle -5.94 [-38.48 30.79] deg *vs* Rim -11.52 [-62.78 20.91] deg. Beta: Vehicle -4.57 [-25.55 24.78] deg *vs* Rim, -3.27 [-32.07 22.82] deg. Gamma: Vehicle 3.97 [-14.33 20.07] deg *vs* Rim

-6.00 [-26.99 10.78] deg. Two-sided Mann-Whitney test: P = 0.152, P = 0.799 and P = 0.020, for each frequency band, respectively. Bottom: Median change in RL. Median ($x10^{-3}$): Theta: Vehicle 2.22 [-15.26 23.23] *vs* Rim 7.65 [-28.40 27.39]. Beta: Vehicle -0.77 [-7.20 12.21] *vs* Rim -0.01 [-12.96 9.20]. Gamma: Vehicle -4.92 [-20.97 6.67] *vs* Rim 2.43 [-7.75 14.65]. Two-sided Mann-Whitney test: P = 0.720, P = 0.697 and P = 0.020 for each frequency band, respectively; n = 79 and n = 77 I_Cells from 13 sessions (7 mice) and 9 sessions (6 mice) for Vehicle and Rim, respectively. Boxplots and data values represent median and 25-75th percentiles ([lower bound upper bound]). n.s., non-significant. Source data and additional distribution characteristics are provided in the **Source Data** file.

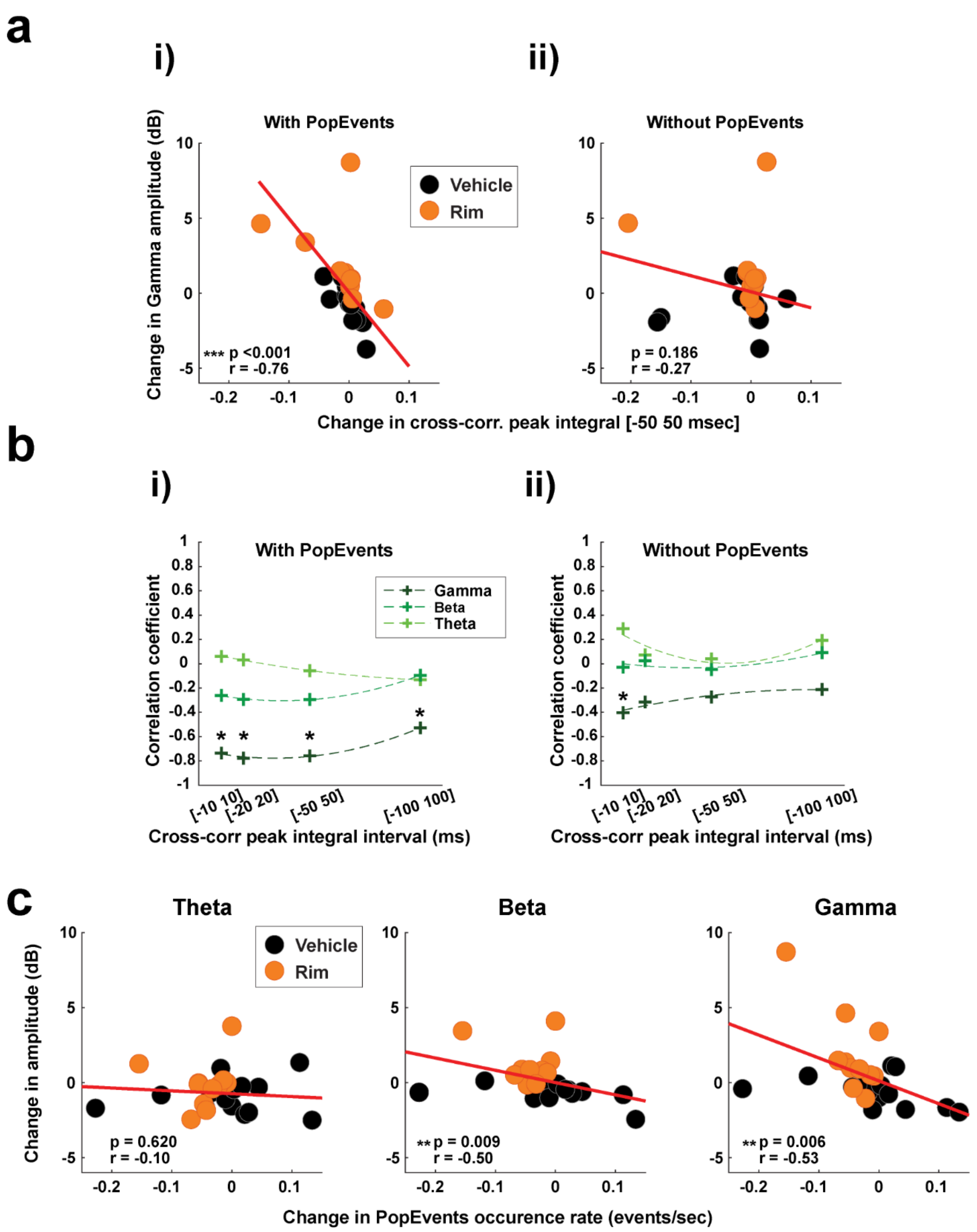

Supplementary Figure 4. Relation between unit co-activation and oscillatory power changes.

(**a**) **i**) Correlation between injection-induced changes in peak integral from cross-correlograms (cross-corr., [-50 50] msec) and changes in gamma power when including population event unit

firing in cross-correlations. Correlation coefficient: r = -0.76; Student's *t* distribution from Spearman's linear correlation coefficients: ***P < 0.001; n = 15 and n= 11 sessions from 8 mice for Vehicle and Rim, respectively. **ii**) Same as (**i**) but excluding population event (PopEvents) unit firing for cross-correlations. Correlation coefficient: r = -0.27; Student's *t* distribution from Spearman's linear correlation coefficients: P = 0.186; n = 15 and n = 10 sessions from 8 and 7 mice for Vehicle and Rim, respectively. (**b**) Correlation coefficients (r) obtained when assessing – across sessions - the link between injection-induced changes in LFP amplitude in the 3 frequency ranges and injection-induced modifications in unit co-activation (peak integral of combined cross-correlograms for all E_Cell pairs using different time windows). Cross-correlations used for peak integrals include (**i**) or exclude (**ii**) firing in PopEvents. **i)** Gamma: [-10 10] msec, r = -0.74; [-20 20] msec, r = -0.78; [-50 50] msec, r = -0.76; [-100 100] msec, r = -0.53; Student's *t* distribution from Spearman's linear correlation coefficients: *P < 0.001, *P < 0.001, *P < 0.001, *P = 0.006 for the 4 intervals, respectively. Theta and Beta: r < 0.30 and P > 0.05. **ii)** Gamma: [-10 10] msec, r = -0.40; [-20 20] msec, r = -0.32; [-50 50] msec, r = -0.27; [-100 100] msec, r = -0.21. Student's *t* distribution from Spearman's linear correlation coefficients: *P = 0.05, P = 0.12, P = 0.19, P = 0.31 for the 4 intervals, respectively. Theta and Beta: r < 0.30 and P > 0.05. n = 15 and n= 11 sessions from 8 mice for Vehicle and Rim, respectively. See **Supplementary Table 2** for statistical details regarding Theta and Beta. (**c**) Relationship between injection-induced changes in PopEvent occurrence rate and changes in Theta, Beta and Gamma amplitude. Vehicle, black dots; Rim, orange dots; red lines, linear regression. Student's *t* distribution from Spearman's linear correlation coefficients: P = 0.620, **P = 0.009 and **P = 0.006, respectively. n = 15 and n = 11 sessions from 8 mice for Vehicle and Rim, respectively. Source data are provided in the **Source Data** file.

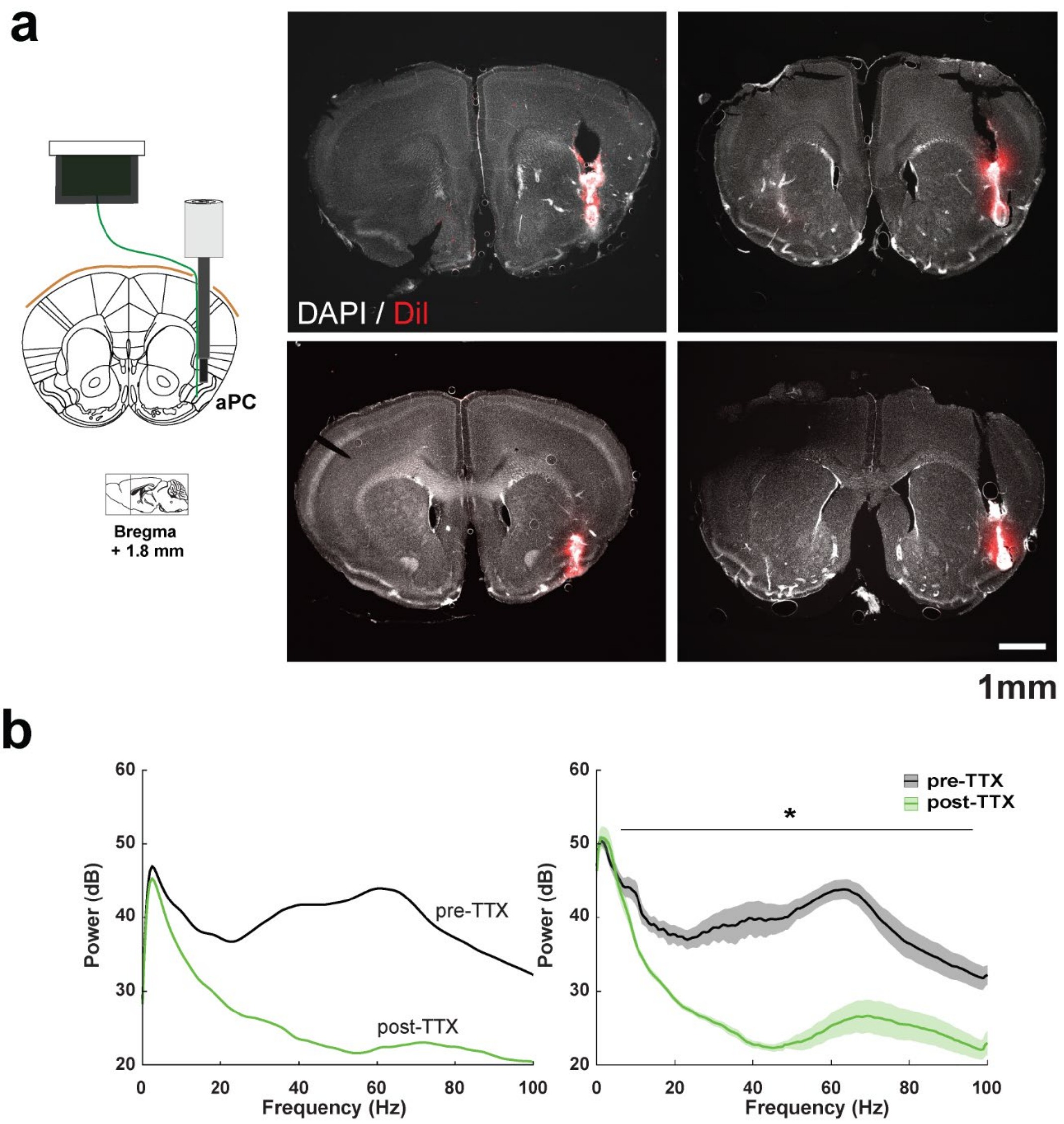

Supplementary Figure 5. Local pharmacological manipulations in the anterior piriform cortex.

(**a**) Left: Schematic of the implant with injection cannula coupled to a tungsten electrode for local field potential recordings (brain section adapted from Paxinos and Watson mouse brain atlas). Right: Histological verification of injection sites in the anterior piriform cortex (aPC). Four brain sections from four different mice implanted with injection cannulas above the aPC. A fluorescent dye (DiI, red) was injected through the injector 1.5 mm below the cannula's tip and the brain tissue was fixed immediately after. Because of its hydrophobic properties, diffusion of DiI beyond the injection site is limited showing the location of the tip of the injector. Scale bar: 1mm. (**b**) Left: example of power spectra pre- and post- local injection of tetrodotoxin (TTX) in aPC. Right: mean change in power spectrum before (gray) or after TTX (green) injections. Two-way ANOVA interaction: ***$P<0.001$. Horizontal line indicates post-hoc student *t* test with Bonferroni correction: *$P<0.05$; n = 4 sessions from 4 mice. Means, bold lines; SEM, shaded areas. Source data are provided as a **Source Data** file.

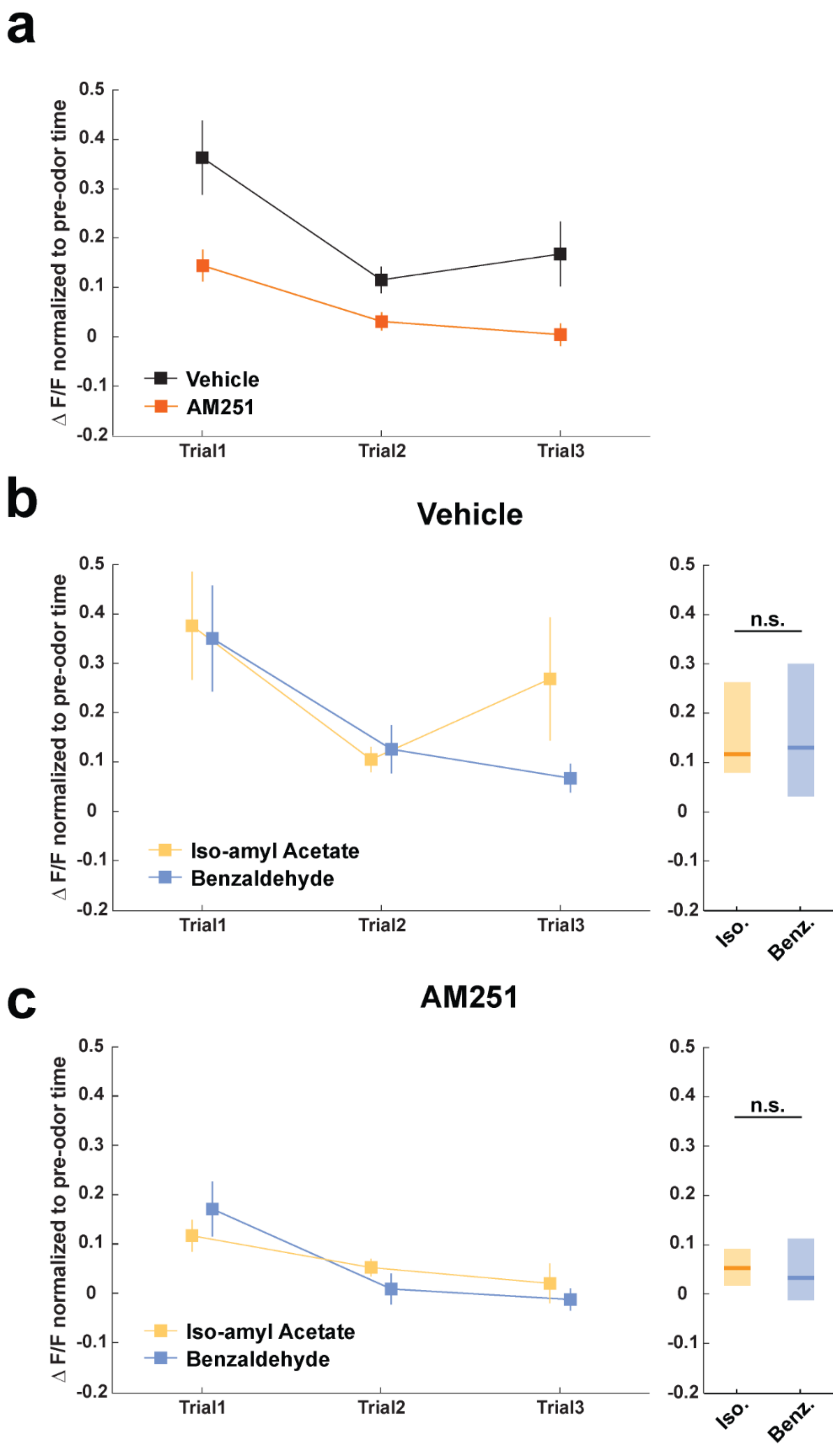
a
Δ F/F normalized to pre-odor time
0.5
0.4
0.3
0.2
0.1
0
-0.1
-0.2
Vehicle
AM251
Trial1
Trial2
Trial3
b
Vehicle
Iso-amyl Acetate
Benzaldehyde
n.s.
Iso.
Benz.
c
AM251
Iso-amyl Acetate
Benzaldehyde
n.s.
Iso.
Benz.

Supplementary Figure 6. Effect of local injections of CB1 receptor antagonist on odor evoked calcium responses.

(**a**) Averaged calcium responses in the anterior piriform cortex (aPC) to Iso-amyl Acetate (Iso.) and Benzaldehyde (Benz.) following local injections of either AM251 or Vehicle. (**b**) Calcium responses following Vehicle local injections in aPC for three successive odor presentation trials (left) and their average (right). Iso. (yellow): 0.115 [0.078 0.260] *vs* Benzaldehyde (blue): 0.129 [0.030 0.298]. Two-sided Wilcoxon's paired signed rank test: P = 0.11; n= 14 hemispheres from 8 mice for Iso. and Benz., respectively. (**c**) Calcium responses following AM251 local injections in the aPC for three successive odor presentation trials (left) and averaged across the three trials (right). Iso.: 0.054 [0.018 0.092] *vs* Benz.: 0.034 [-0.011 0.113]. Two-sided Wilcoxon's paired signed rank test: P = 0.88; n= 20 hemispheres from 10 mice for Iso. and Benz, respectively. Boxplots and data values represent median and 25-75th percentiles ([lower bound upper bound]). n.s., non-significant. Source data and additional distribution characteristics are provided in the **Source Data** file.

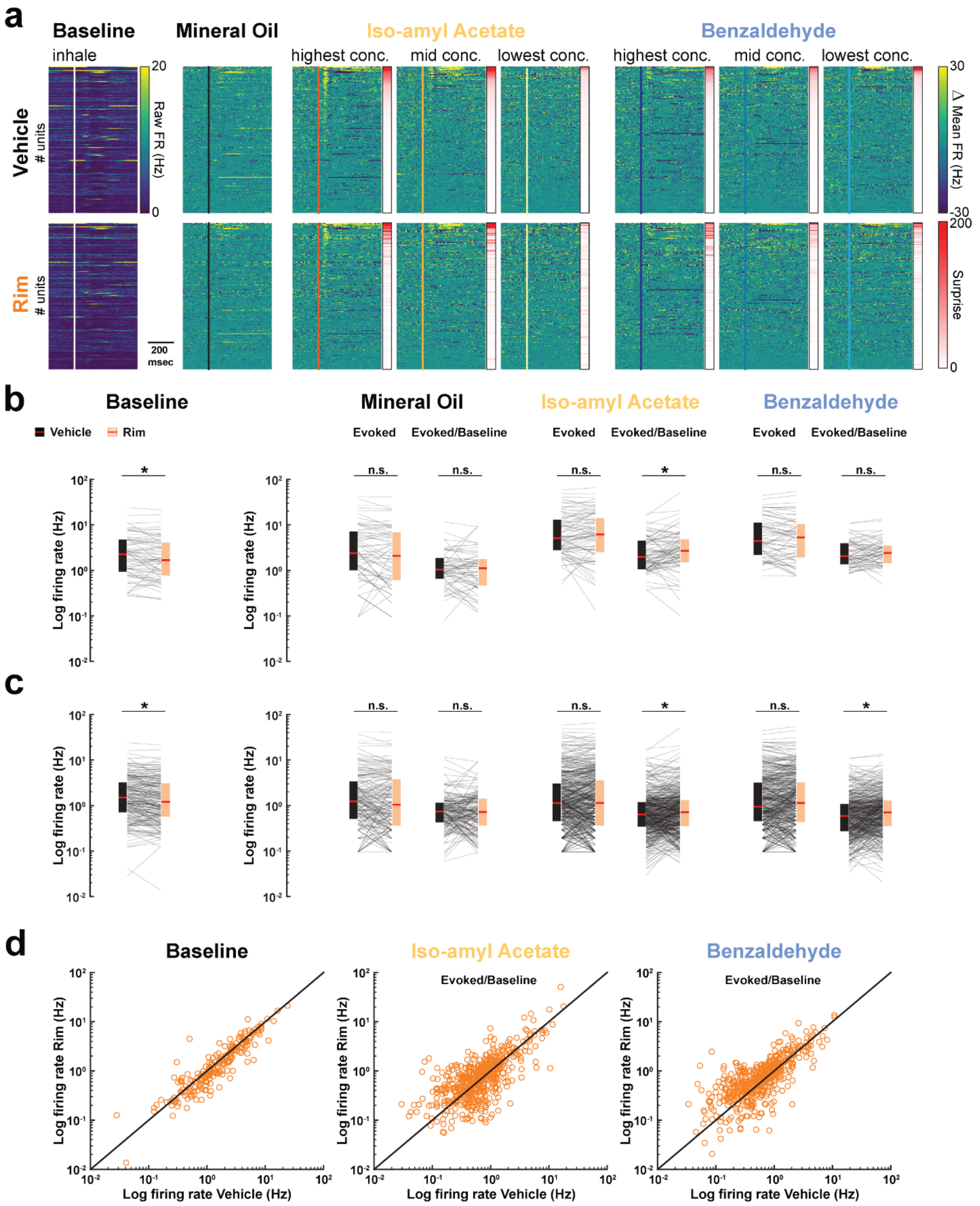

Supplementary Figure 7. Effect of systemic injection of CB1 receptor antagonist on odor evoked activity in the anterior piriform cortex.

(**a**) Trial-averaged firing rates of E_Cells aligned to first breaths in the presence of stimulus or in the baseline period following systemic injection of Vehicle (top) and Rim (bottom). Activity is aligned to the first inhale onset (vertical lines) in the window considered (baseline or stimulus, 2 sec in duration). Baseline window starts 3 sec before each trial. Mineral Oil-induced firing rates are computed by subtracting baseline firing rates while odor-induced firing rates are computed by subtracting Mineral Oil-induced firing rates (to control for expectation and valve clicks). Iso-amyl Acetate (yellow) and Benzaldehyde (blue) are randomly presented at 3 distinct concentrations (highest, middle, lowest). Surprise represents summed spiking probability in presence of the stimulus according to the Poisson distributions derived from the baseline firing rate of each cell (See **Methods**). n = 207 E_Cells from 3 sessions across 2 animals for Vehicle and Rim. (**b**) Log firing rates from E_Cells that significantly respond to at least one of the 6 odor stimuli (n = 70). For mineral oil pairs, odor-responsive units that have firing rates >0.1 Hz after mineral oil presentation (n=63) in both Vehicle and Rim conditions. Unit-odor pairs (n= 80 for iso-amyl acetate and n=66 for benzaldehyde) are determined by detecting significant odor responses for a cell (surprise test, See **Methods**) for the 3 different concentrations of an odor. Each odor concentration is treated separately and then unit-concentration responses are pooled, meaning a cell can contribute up to 3 points in a plot if it responds to all 3 concentrations of a single odor. Evoked firing refers to raw spike rates, and evoked/baseline refers to divisive normalization by baseline rates. Baseline: Vehicle 2.237 [0.980 4.547] Hz *vs* Rim 1.674 [0.803 3.887] Hz. Two-sided Wilcoxon's test: P = 0.013. Mineral Oil Evoked: Vehicle 2.381 [1.048 6.828] Hz *vs* Rim 2.095 [0.634 6.524] Hz. Two-sided Wilcoxon's test: P = 0.009. Odors: Iso-amyl acetate Evoked - Vehicle 5.123 [2.904 12.429] Hz *vs* Rim 6.156 [2.607 13.524] Hz, Two-sided Wilcoxon's test: P = 0.571. Iso-amyl Acetate Evoked/Baseline - Vehicle 1.997 [1.107 4.326] *vs* Rim 2.700 [1.579 4.626], Two-sided Wilcoxon's test: P = 0.012. Benzaldehyde Evoked - Vehicle 4.429 [2.286 10.676] Hz *vs* Rim 5.271 [2.010 10.044] Hz, Two-sided Wilcoxon's test: P = 0.256. Benzaldehyde Evoked/Baseline - Vehicle 2.050 [1.421 3.787] *vs* Rim 2.412 [1.500 3.373], Two-sided Wilcoxon's test: P = 0.175. E_Cells recorded from 3 sessions across 2 mice. (**c**) Log firing rates in all recorded E_Cells with firing rates >0.1 Hz in each tested condition. Baseline (n = 207): Vehicle 1.497 [0.980 4.547] Hz *vs* Rim 1.211 [0.803 3.887] Hz. Two-sided Wilcoxon's test: P < 0.001. Mineral Oil (n = 175) Evoked: Vehicle 1.238 [0.532 3.245] Hz *vs* Rim 1.053 [0.381 3.619] Hz, Two-sided Wilcoxon's test: P = 0.054. Mineral Oil Evoked/Baseline: Vehicle 0.743 [0.447 1.107] *vs* Rim 0.719 [0.379 1.355], Two-sided Wilcoxon's test: P = 0.565. Odors: Iso-amyl acetate (n=507) Evoked - Vehicle 1.143 [0.476 2.905] Hz *vs* Rim 1.143 [0.381 3.429] Hz, Two-sided Wilcoxon's test: P = 0.680. Iso-amyl acetate Evoked/Baseline - Vehicle 0.640 [0.361 1.134] *vs* Rim 0.714 [0.368 1.258], Two-sided Wilcoxon's test: P < 0.001. Benzaldehyde (n=477) Evoked - Vehicle 0.952 [0.476 3.048] Hz *vs* Rim 1.143 [0.451 3.129] Hz, Two-sided Wilcoxon's test: P = 0.224. Benzaldehyde Evoked/Baseline - Vehicle 0.586 [0.286 1.039] *vs* Rim 0.708 [0.368 1.229], Two-sided Wilcoxon's test: P < 0.001. (**d**) Distribution of log firing rates for E_Cells between Vehicle and Rim in Baseline, Iso-amyl Acetate and Benzaldehyde. N = 207 E_Cells recorded from 3 sessions across 2 mice. Boxplots and data values represent median and 25-75th percentiles ([lower bound upper bound]). n.s., non-significant. Source data and additional distribution characteristics are provided in the **Source Data** file.

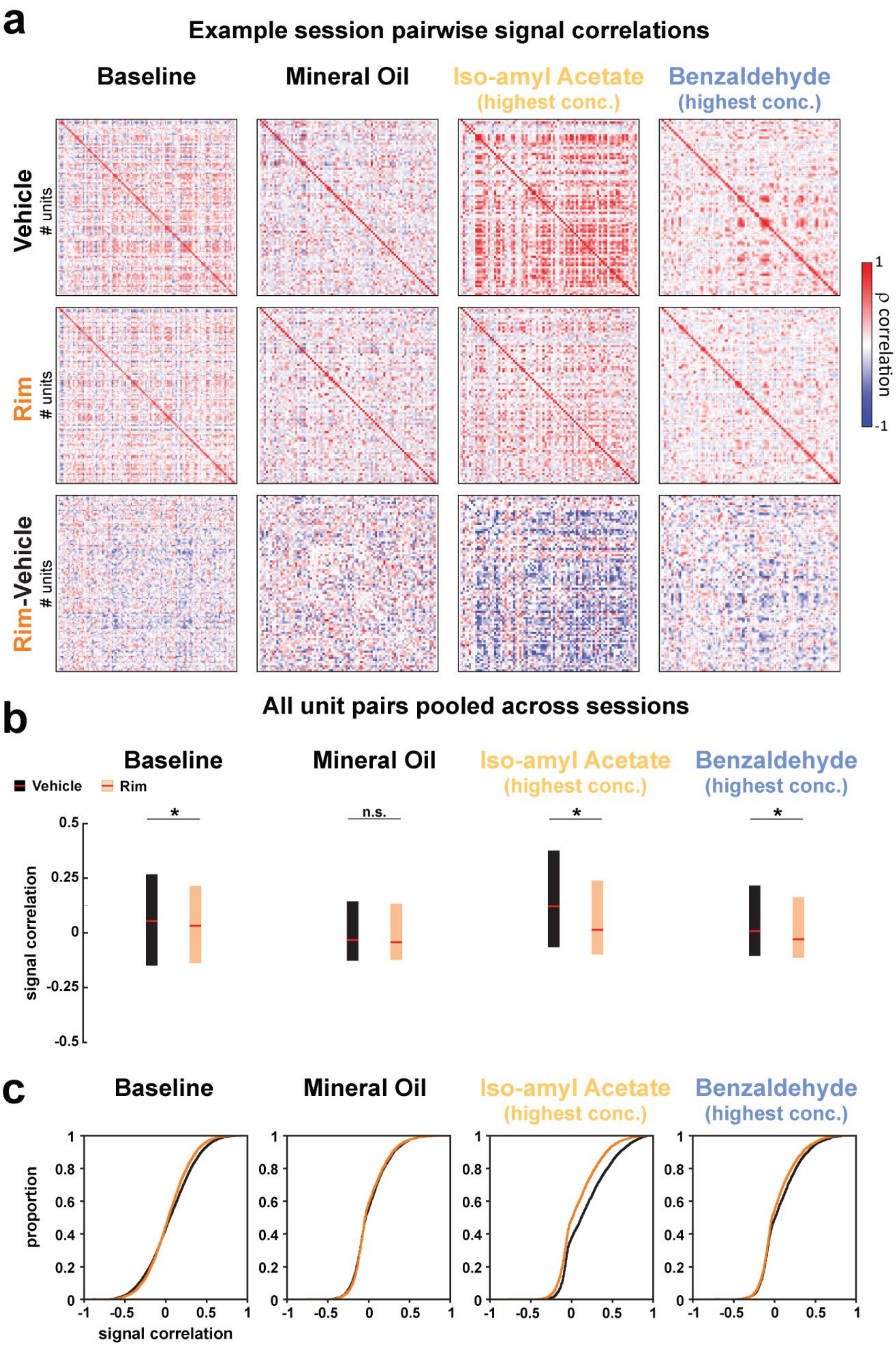

a
Example session pairwise signal correlations
Baseline
Mineral Oil
Iso-amyl Acetate
(highest conc.)
Benzaldehyde
(highest conc.)
Vehicle
units
Rim
units
Rim-Vehicle
units
1
ρ correlation
-1
b
All unit pairs pooled across sessions
Baseline
Mineral Oil
Iso-amyl Acetate
(highest conc.)
Benzaldehyde
(highest conc.)
Vehicle
Rim
*
n.s.
*
*
0.5
0.25
0
-0.25
-0.5
signal correlation
c
Baseline
Mineral Oil
Iso-amyl Acetate
(highest conc.)
Benzaldehyde
(highest conc.)
proportion
1
0.8
0.6
0.4
0.2
0
-1
-0.5
0
0.5
1
signal correlation

Supplementary Figure 8. Effect of systemic injection of CB1 receptor antagonist on odor evoked signal correlation in aPC.

(**a**) From one example session, raw pairwise signal correlations (E_Cells only) computed from the 350 msec following first breaths in the presence of stimulus or in the baseline period after systemic injection of Vehicle (top) and Rim (middle). The difference between Vehicle and Rim is also plotted (bottom). Pairwise signal correlations are shown for units that respond to the highest concentration of either Iso-amyl Acetate (Iso., yellow) and Benzaldehyde (Benz., blue) and have firing rates >0.1 Hz. (**b**) Unit-pair signal correlations from all E_Cells with firing rates >0.1 Hz across all sessions and (**c**) cumulative distribution function of the signal correlations. Baseline: Vehicle 0.054 [-0.145 0.263] *vs* Rim 0.033 [-0.133 0.210]; Mineral Oil: Vehicle -0.033 [-0.124 0.140] *vs* Rim -0.042 [-0.118 0.128]; Iso-amyl Acetate: Vehicle 0.122 [-0.062 0.372] *vs* Rim 0.014 [-0.093 0.235]; Benzaldehyde: Vehicle 0.009 [-0.101 0.212] *vs* Rim -0.029 [-0.108 0.158]. Two-sided Wilcoxon's test: $P < 0.001$; $P = 0.178$; $P < 0.001$; $P < 0.001$. $n = 8539$; $n = 5721$; $n = 5329$ and $n = 4365$ unit-pairs from 3 sessions across 2 mice for Baseline, Mineral Oil, Iso. and Benz., respectively. Boxplots and data values represent median and 25-75th percentiles ([lower bound upper bound]). n.s., non-significant. Source data and additional distribution characteristics are provided in the **Source Data** file.

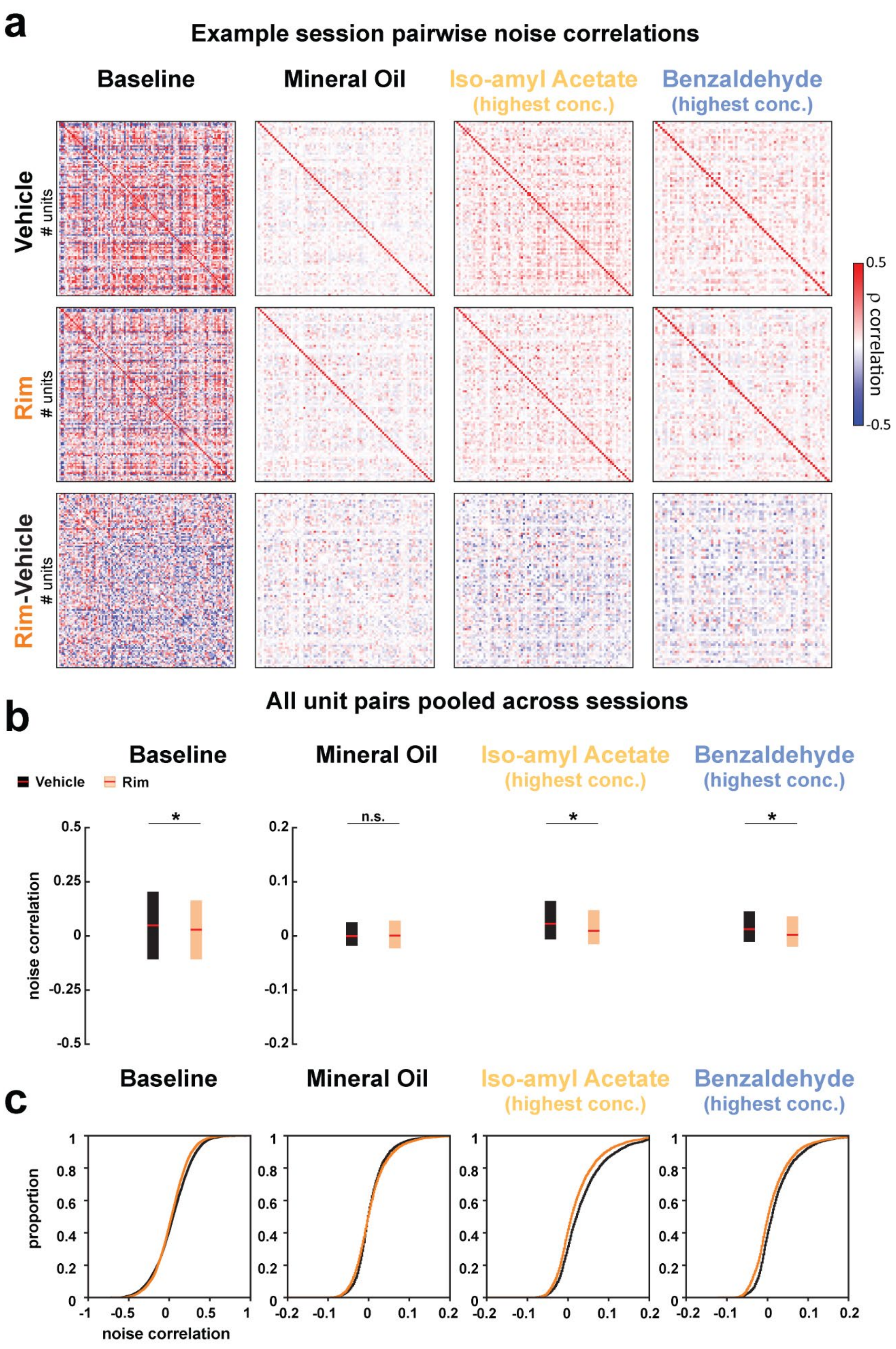
a
Example session pairwise noise correlations
Baseline
Mineral Oil
Iso-amyl Acetate
(highest conc.)
Benzaldehyde
(highest conc.)
Vehicle
units
Rim
units
Rim-Vehicle
units
0.5
ρ correlation
-0.5
b
All unit pairs pooled across sessions
Baseline
Mineral Oil
Iso-amyl Acetate
(highest conc.)
Benzaldehyde
(highest conc.)
Vehicle
Rim
*
n.s.
*
*
noise correlation
c
Baseline
Mineral Oil
Iso-amyl Acetate
(highest conc.)
Benzaldehyde
(highest conc.)
proportion
noise correlation

Supplementary Figure 9. Effect of systemic injection of CB1 receptor antagonist on odor evoked noise correlations.

(**a**) From one example session, raw pairwise noise correlations (E_Cells only) computed from the 350 msec following first breaths in the presence of stimulus or in the baseline period after systemic injection of Vehicle (top) and Rim (middle). The difference between Vehicle and Rim is also plotted (bottom). Pairwise noise correlations are shown for units that respond to the highest concentration of either Iso-amyl Acetate (Iso., yellow) and Benzaldehyde (Benz., blue) and have firing rates >0.1 Hz. (**b**) Unit-pair noise correlations from all E_Cells with firing rates >0.1 Hz across all sessions and (**c**) cumulative distribution function of the noise correlations. Baseline: Vehicle 0.0481 [-0.104 0.200] *vs* Rim 0.028 [-0.104 0.160]; Mineral Oil: Vehicle -0.0002 [-0.017 0.023] *vs* Rim 0.0003 [-0.021 0.026]; Iso-amyl Acetate: Vehicle 0.0224 [-0.005 0.063] *vs* Rim 0.0095 [-0.013 0.046]; Benzaldehyde: Vehicle 0.0122 [-0.009 0.044] *vs* Rim 0.0023 [-0.018 0.034]. Two-sided Wilcoxon's test: $P < 0.001$; $P = 0.538$; $P < 0.001$; $P < 0.001$. n = 8539; n = 5721; n = 5329 and n = 4365 unit-pairs from 3 sessions across 2 mice for Baseline, Mineral Oil, Iso. and Benz., respectively. Boxplots and data values represent median and 25-75th percentiles ([lower bound upper bound]). n.s., non-significant. Source data are provided in the **Source Data** file.

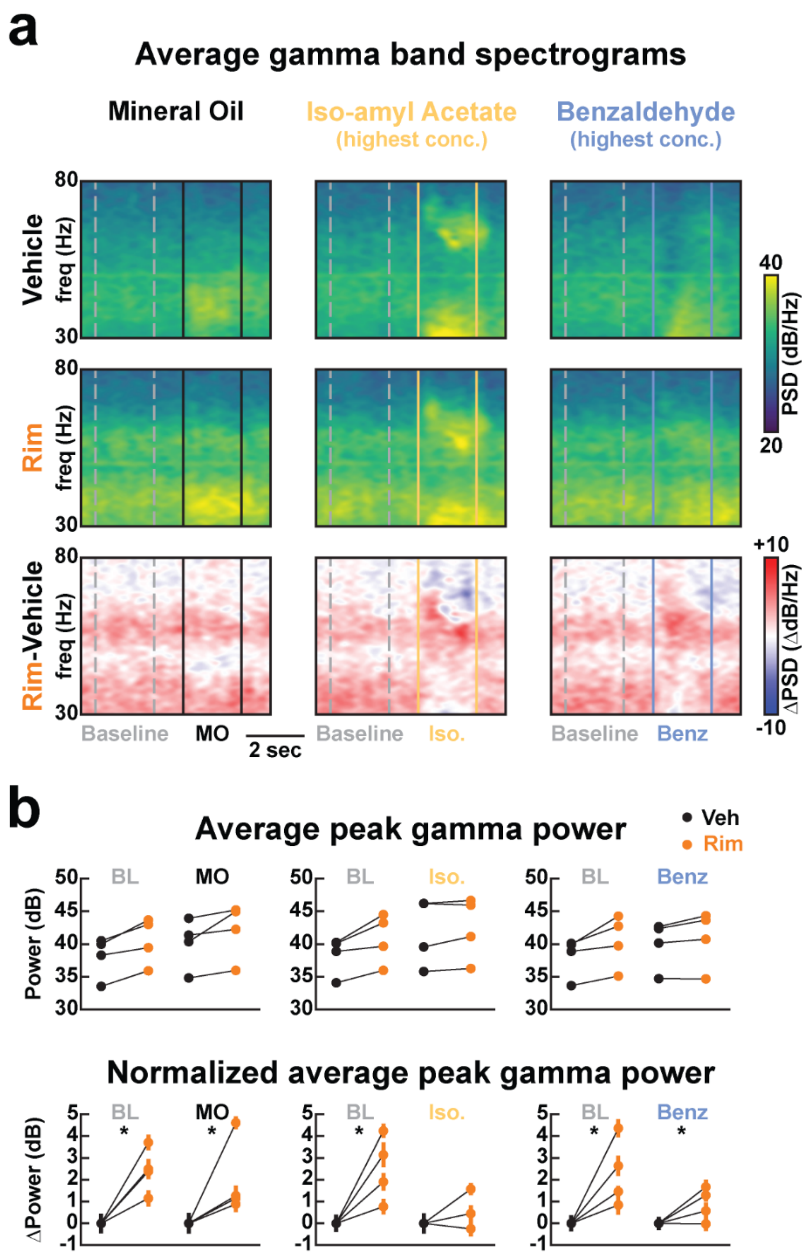

Supplementary Figure 10. Effect of systemic injection of CB1 receptor antagonist gamma oscillations upon olfactory stimulations in the olfactometer.

(**a**) Grand average gamma band spectrograms (averaged across 4 sessions, 30 trials per session) after systemic injection of Vehicle (top) or Rimonabant (Rim, middle), along with their difference (Rim-Vehicle, bottom). Spectrograms are aligned to olfactometer-controlled presentation of mineral oil (MO, left), or the highest concentration of iso-amyl acetate (Iso., left), or the highest concentration of benzaldehyde (Benz., right). Baseline periods (2-second windows 1 sec before stimulation) are demarcated in grey dashed lines, while odor presentation periods are solid lines of the appropriate color. (**b**) Top: Trial-averaged peak gamma powers from the baseline (BL) or

odor presentation periods after systemic injection of Vehicle (Veh) or Rim. Each pair of points (and standard error bars) is derived from one session (4 sessions in total). Bottom: the same as above but normalized by subtracting the mean peak gamma power from the Veh condition in each session. Significance assessed by shuffling Vehicle and Rim trial labels 1000 times and re-computing metrics with surrogate data. * indicates that $p<0.001$. Mean differences in dB (Rim-Veh) for the BL period were 2.43, 2.51, and 2.32 for MO, Iso. and Benz. respectively. The max differences seen across 1000 shuffles were 1.46, 1.29, and 1.19 respectively. Mean differences in dB (Rim-Veh) for the odor-presentation periods were 1.98, 0.55, and 0.88 for MO, Iso., and Benz. respectively. The max differences seen across 1000 shuffles were 1.42, 0.66, and 0.78 respectively. Source data are provided in the **Source Data** file.

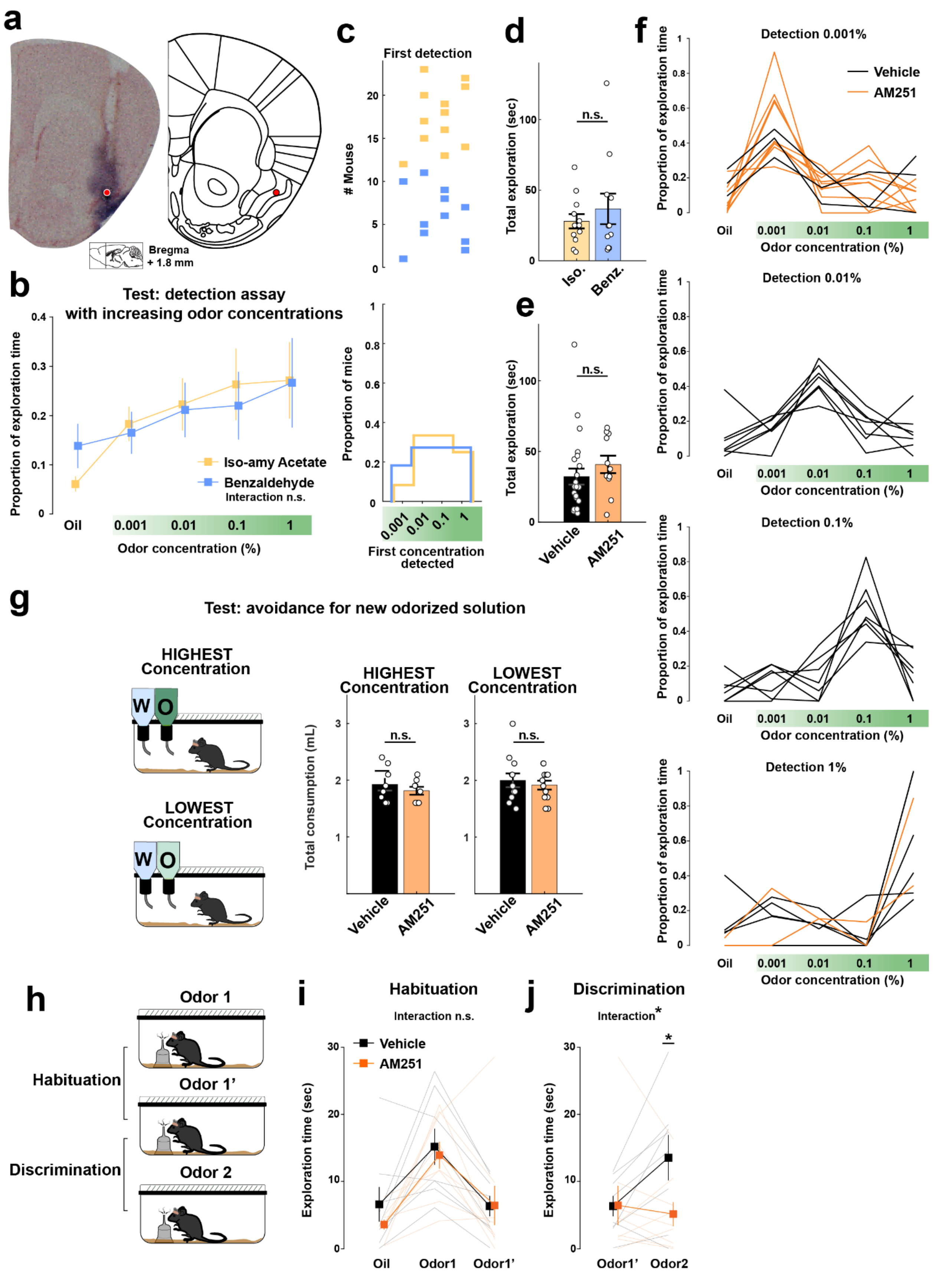

a
Bregma + 1.8 mm
b
Test: detection assay with increasing odor concentrations
Proportion of exploration time
Iso-amy Acetate
Benzaldehyde
Interaction n.s.
Oil
0.001
0.01
0.1
1
Odor concentration (%)
c
First detection
Mouse
Proportion of mice
First concentration detected
d
Total exploration (sec)
n.s.
Iso.
Benz.
e
Total exploration (sec)
n.s.
Vehicle
AM251
f
Detection 0.001%
Detection 0.01%
Detection 0.1%
Detection 1%
Vehicle
AM251
Proportion of exploration time
Odor concentration (%)
g
Test: avoidance for new odorized solution
HIGHEST Concentration
LOWEST Concentration
W
O
Total consumption (mL)
n.s.
h
Odor 1
Odor 1'
Odor 2
Habituation
Discrimination
i
Habituation
Interaction n.s.
Vehicle
AM251
Exploration time (sec)
Oil
Odor1
Odor1'
j
Discrimination
Interaction*
Odor1'
Odor2

Supplementary Figure 11. Effect of local injection of CB1 receptor antagonist on olfactory behaviors.

(**a**) Left: histological coronal section with an injection of pontamine sky blue and the track of a cannula in the aPC. Right: schematic section adapted from Paxinos and Watson mouse brain atlas. Red dot represents the injection site. (**b**) Proportion of exploration time spent sniffing mineral oil and increasing concentration of Benzaldehyde (Benz., blue) and Iso-amyl Acetate (Iso., yellow) odors following local injections of Vehicle in anterior piriform cortex (aPC). Mean, square symbols; SEM, outer lines. Two-way ANOVA interaction: P = 0.916. (**c**) Top: distribution of individual mice according to the concentration of Benzaldehyde (blue) or Iso-amyl Acetate (yellow) they first detected. Bottom: proportions of mice with first detection at specified concentration. Odor concentration 0.001%, 0.18 in Benz. *vs* 0.08 in Iso.; concentration 0.01%, 0.27 *vs* 0.33; concentration 0.1%, 0.27 *vs* 0.33; concentration 1%, 0.27 *vs* 0.25; Two-sided Chi square tests between Benz. and Iso.: P > 0.05 for all concentrations; n = 11 and n = 12 mice for Benz. and Iso., respectively. See **Supplementary Table 2** for statistic details. (**d**) Total exploration time within the 5 trials comparing Iso. and Benz. Iso. mean 27.95, SEM ± 4.99 sec *vs* Benz. mean 36.75, SEM ± 10.83 sec. Two-sided Mann-Whitney test: P = 1. (**e**) Same as (**d**) but comparing Vehicle and AM251. Vehicle (mean ± SEM) 32.16 ± 5.74 sec *vs* AM251 40.75 ± 6.18 sec. Two-sided Mann-Whitney test: P = 0.162. (**f**) Proportion of exploration time (i.e. spent sniffing the nozzle) for individual mice with each panel representing a group of mice with the same odor concentration threshold (0.001%, 0.01%, 0.1% or 1%). (**g**) Total liquid consumption from water and the highest or the lowest concentration of a new odorized solution (see **Methods**). Highest concentration (mean ± SEM): Vehicle 1.93 ± 0.11 mL *vs* AM251 1.81 ± 0.07 mL. Lowest concentration: Vehicle 2 ± 0.12 mL *vs* AM251 1.92 ± 0.08 mL. Two-sided Mann-Whitney test: P = 0.556 and P = 0.793, for highest and lowest concentration respectively. (**h**) Schematic of the experimental design used to test odor habituation and odor discrimination performances. The duration mice spent sniffing the nozzle (exploration time) is quantified when the same odor is presented twice (Odor 1 → Odor 1’, odor habituation) and when a novel odor is presented after the familiar odor (Odor1’ → Odor 2, odor discrimination). (**i**) Exploration time for odor habituation and for odor discrimination (**j**) following Vehicle (black) or AM251 (orange) local injections in the aPC. Two-way ANOVA interaction P = 0.714 and P = 0.012 for odor habituation and discrimination, respectively; post-hoc student *t* test with Bonferroni correction: *P = 0.05; n = 8 and n = 9 mice for Vehicle and AM251, respectively. n.s., non-significant. Source data are provided as a **Source Data** file.

Supplementary Tables.

Supplementary Table 1

Figure	n	Analysis (posthoc)	Factors analyzed		Degrees of freedom - zval	P values
1e	10 sessions (7 mice)	Wilcoxon signed rank	pre *vs* post Rim		-	0.002
1f	15 sessions (8 mice) - 10 sessions (7 mice)	Mann-Whitney	Vehicle *vs* Rim		2.413	0.016
1g	6764 pairs (17 sessions) - 5099 pairs (11 sessions)	Mann-Whitney	Vehicle *vs* Rim		9.600	<0.001
1h	6015 pairs (15 sessions) - 4787 pairs (10 sessions)	Mann-Whitney	Vehicle *vs* Rim		-0.274	0.784
2b	17 sessions (9 mice) - 11 sessions (8 mice)	Two-way ANOVA (post-hoc Bonferroni)	Vehicle *vs* Rim	Interaction	F (15, 390) = 8.594	<0.001
				Frequency effect	F (15, 390) = 3.832	<0.001
				Treatment effect	F (1, 26) = 8.971	0.006
				from 24 to 54Hz (post-hoc)		<0.05
2c	17 sessions (9 mice) - 11 sessions (8 mice)	Mann-Whitney	Vehicle *vs* Rim - Theta		-2.023	0.043
			Vehicle *vs* Rim - Beta		-4.046	<0.001
			Vehicle *vs* Rim - Gamma		3.152	0.002
2e	456 units (17 sessions) - 305 units (11 sessions)	Mann-Whitney	Vehicle *vs* Rim - Angular distance -Theta		-0.378	0.705
			Vehicle *vs* Rim - Resultant length -Theta		0.507	0.612
			Vehicle *vs* Rim - Angular distance - Beta		-0.012	0.990
			Vehicle *vs* Rim - Resultant length -Beta		-1.386	0.166
			Vehicle *vs* Rim - Angular distance -Gamma		-1.173	0.241
			Vehicle *vs* Rim - Resultant length -Gamma		-8.097	<0.001
3b	14 sessions (8 mice) - 14 sessions (8 mice)	Two-way ANOVA (post-hoc Bonferroni)	Vehicle *vs* AM251	Interaction	F (15, 390) = 3.212	<0.001
				Frequency effect	F (15,390) = 5.854	<0.001
				Treatment effect	F (1, 26) = 1.470	0.236
				from 30 to 35 Hz (post-hoc)		<0.05
3f	6 hemispheres (8 mice) - 20 hemispheres (10 mice	Mann-Whitney	Vehicle *vs* AM251		3.089	0.002
4c	4 hemispheres (8 mice) - 20 hemispheres (10 mice	Mann-Whitney	Vehicle *vs* AM251	Mineral Oil	-0.577	0.564
				Odors	3.315	<0.001
4f	40 units (3 sessions)	Wilcoxon signed rank	Vehicle *vs* Rim	Mineral Oil	-1.223	0.221
				Odors	-6.393	<0.001
4g	254 unit-pairs (3 sessions)	Wilcoxon signed rank	Vehicle *vs* Rim	Mineral Oil	-3.533	0.660
				Odors	-0.440	<0.001
5b	23 mice - 11 mice	Two-way ANOVA (post-hoc Bonferroni)	Vehicle *vs* AM251	Interaction	F (4, 128) = 4.342	0.003
				Concentration effect	F (4, 128) = 4.731	0.001
				Treatment effect	F (1, 32) = 0.050	0.824
				Oil		>0.999
				0.001%		0.016
				0.01%		0.803
				0.1%		0.802
				1%		>0.999
5c	23 mice - 11 mice	Chi square	Vehicle *vs* AM251	0.001% vs other concentrations	3.926	<0.001
				0.01% vs other concentrations	2.053	0.040
				0.1% vs other concentrations	2.053	0.040
				1% vs other concentrations	0.567	0.571
5d	8 mice - 8 mice	Two-way ANOVA (post-hoc Bonferroni)	Vehicle *vs* AM251	Interaction	F (1, 14) = 0.816	0.382
				Bottle effect	F (1, 14) = 22.37	<0.001
				Treatment effect	F (1, 14) = 0.751	0.401
5e	12 mice - 12 mice	Two-way ANOVA (post-hoc Bonferroni)	Vehicle *vs* AM251	Interaction	F (1, 22) = 4.884	0.038
				Bottle effect	F (1, 22) = 1.439	0.243
				Treatment effect	F (1, 22) = 0.333	0.570
				Water *vs* Odor-Vehicle		0.965
				Water *vs* Odor-Antago		0.049

Supplementary Table 2 (PART 1/2)

Figure Supp	n	Analysis (posthoc)	Factors analyzed		Degrees of freedom - zval	P values
2a	393 units (11 sessions)	Wilcoxon signed rank	pre *vs* post Rim		-1.724	0.085
2b	645 units (17 sessions) - 393 units (11 sessions)	Mann-Whitney	Vehicle *vs* Rim		-3.147	0.002
	456 units (17 sessions) - 305 units (11 sessions)	Mann-Whitney	Vehicle *vs* Rim	[0 10]	1.610	0.107
				[10 50]	-3.580	<0.001
2c	78 units (9 sessions)	Wilcoxon signed rank	pre *vs* post Rim		-2.089	0.037
2d	80 units (13 sessions) - 78 units (9 sessions)	Mann-Whitney	Vehicle *vs* Rim		-0.395	0.693
	79 units (13 sessions) - 77 units (9 sessions)	Mann-Whitney	Vehicle *vs* Rim	[0 10]	-0.223	0.823
				[10 50]	0.308	0.757
3c	79 units (13 sessions) - 77 units (9 sessions)	Mann-Whitney	Vehicle *vs* Rim - Angular distance -Theta		1.432	0.152
			Vehicle *vs* Rim - Resultant length -Theta		-0.358	0.720
			Vehicle *vs* Rim - Angular distance - Beta		0.255	0.799
			Vehicle *vs* Rim - Resultant length -Beta		0.390	0.700
			Vehicle *vs* Rim - Angular distance -Gamma		2.318	0.020
			Vehicle *vs* Rim - Resultant length -Gamma		-3.102	0.002
4a	15 sessions (8 mice) - 11 sessions (8 mice)	Linear Regression analysis	correlation coefficient with PopEvents	[-50 50] - Gamma	-	<0.001
	15 sessions (8 mice) - 10 sessions (7 mice)		correlation coefficient without PopEvents	[-50 50] - Gamma	-	0.186
4b	15 sessions (8 mice) - 11 sessions (8 mice)	Linear Regression analysis	correlation coefficient with PopEvents	[-10 10] - Gamma	-	<0.001
				[-10 10] - Beta	-	0.196
				[-10 10] - Theta	-	0.771
				[-20 20] - Gamma	-	<0.001
				[-20 20] - Beta	-	0.144
				[-20 20] - Theta	-	0.880
				[-50 50] - Gamma	-	<0.001
				[-50 50] - Beta	-	0.143
				[-50 50] - Theta	-	0.776
				[-100 100] - Gamma	-	0.006
				[-100 100] - Beta	-	0.639
				[-100 100] - Theta	-	0.518
	15 sessions (8 mice) - 10 sessions (7 mice)	Linear Regression analysis	correlation coefficient without PopEvents	[-10 10] - Gamma	-	0.047
				[-10 10] - Beta	-	0.890
				[-10 10] - Theta	-	0.162
				[-20 20] - Gamma	-	0.125
				[-20 20] - Beta	-	0.905
				[-20 20] - Theta	-	0.731
				[-50 50] - Gamma	-	0.186
				[-50 50] - Beta	-	0.821
				[-50 50] - Theta	-	0.850
				[-100 100] - Gamma	-	0.309
				[-100 100] - Beta	-	0.665
				[-100 100] - Theta	-	0.357
4c	15 sessions (8 mice) - 11 sessions (8 mice)	Linear Regression analysis	Theta change *vs* PopEvents change		-	0.620
			Beta change *vs* PopEvents change		-	0.009
			Gamma change *vs* PopEvents change		-	0.006
5b	4 sessions (4 mice)	Two-way ANOVA (post-hoc Bonferroni)	pre *vs* post TTX	Interaction	F (15, 45) = 11.46	<0.001
				Frequency effect	F (15,45)=24.66	<0.001
				Treatment effect	F (1,3) = 133.4	P=0.001
				from 6 to 100Hz (post-hoc)		<0.05
6b	14 hemispheres (8 mice)	Wilcoxon signed rank	Iso-amyl Acetate vs Benzaldehyde		-	0.107
6c	20 hemispheres (10 mice)	Wilcoxon signed rank	Iso-amyl Acetate vs Benzaldehyde		-	0.881

Supplementary Table 2 (PART 2/2)

7b	70 units (3 sessions)	Wilcoxon signed rank	Baseline - Vehicle *vs* Rim		-2.496	0.013
	63 units (3 sessions)	Wilcoxon signed rank	Mineral Oil - Vehicle *vs* Rim	Evoked	-2.602	0.009
				Evoked/Baseline	-1.047	0.295
	80 units (3 sessions)	Wilcoxon signed rank	Iso. - Vehicle *vs* Rim	Evoked	-0.566	0.571
				Evoked/Baseline	-2.518	0.012
	66 units (3 sessions)	Wilcoxon signed rank	Benz. - Vehicle *vs* Rim	Evoked	-1.137	0.256
				Evoked/Baseline	-1.357	0.175
7c	207 units (3 sessions)	Wilcoxon signed rank	Baseline - Vehicle *vs* Rim		-5.001	<0.001
	175 units (3 sessions)	Wilcoxon signed rank	Mineral Oil - Vehicle *vs* Rim	Evoked	-1.928	0.054
				Evoked/Baseline	-0.575	0.565
	507 units (3 sessions)	Wilcoxon signed rank	Iso. - Vehicle *vs* Rim	Evoked	-0.405	0.680
				Evoked/Baseline	-3.293	<0.001
	477 units (3 sessions)	Wilcoxon signed rank	Benz. - Vehicle *vs* Rim	Evoked	-1.216	0.224
				Evoked/Baseline	-6.220	<0.001
8b	8539 unit-pairs (3 sessions)	Wilcoxon signed rank	Baseline - Vehicle *vs* Rim		-5.815	<0.001
	5721 unit-pairs (3 sessions)	Wilcoxon signed rank	Mineral Oil - Vehicle *vs* Rim		-1.346	0.178
	5329 unit-pairs (3 sessions)	Wilcoxon signed rank	Iso. - Vehicle *vs* Rim		-21.255	<0.001
	4365 unit-pairs (3 sessions)	Wilcoxon signed rank	Benz. - Vehicle *vs* Rim		-6.999	<0.001
9b	8539 unit-pairs (3 sessions)	Wilcoxon signed rank	Baseline - Vehicle *vs* Rim		-8.423	<0.001
	5721 unit-pairs (3 sessions)	Wilcoxon signed rank	Mineral Oil - Vehicle *vs* Rim		-0.616	0.538
	5329 unit-pairs (3 sessions)	Wilcoxon signed rank	Iso. - Vehicle *vs* Rim		-13.000	<0.001
	4365 unit-pairs (3 sessions)	Wilcoxon signed rank	Benz. - Vehicle *vs* Rim		-10.199	<0.001
10b	30 trials (4 sessions)	permutation test to shuffle trial labels (1000 times)	Baseline Mineral Oil - Vehicle *vs* Rim		-	<0.001
			Mineral Oil - Vehicle *vs* Rim		-	<0.001
			Baseline Iso. - Vehicle *vs* Rim		-	<0.001
			Iso. - Vehicle vs Rim		-	>0.001
			Baseline Benz. - Vehicle *vs* Rim		-	<0.001
			Benz. - Vehicle *vs* Rim		-	<0.001
11b	11 mice - 12 mice	Two-way ANOVA (post-hoc Bonferroni)	Iso-amyl Acetate *vs* Benzaldehyde	Interaction	F (4, 84) = 0.238	0.916
				Concentration effect	F (4, 84) = 1.996	0.103
				Treatment effect	F (1, 21) = 0.144	0.708
11c	11 mice - 12 mice	Chi square	Iso-amyl Acetate vs Benzaldehyde	0.001% vs other concentrations	0.701	0.484
				0.01% vs other concentrations	0.316	0.752
				0.1% vs other concentrations	0.509	0.611
				1% vs other concentrations	0.124	0.901
11d	11 mice - 12 mice	Mann-Whitney	Iso-amyl Acetate vs Benzaldehyde		0.000	1.000
11e	23 mice - 11 mice	Mann-Whitney	Vehicle *vs* AM251		-1.399	0.162
11g	8 mice - 8 mice	Mann-Whitney	Vehicle *vs* AM251	Highest concentration	0.588	0.556
	12 mice - 12 mice			Lowest concentration	0.262	0.793
11i	8 mice - 9 mice	Two-way ANOVA (post-hoc Bonferroni)	Vehicle *vs* AM251	Interaction	F (2, 30) = 0.341	0.714
				Odor effect	F (2, 30) = 14.45	<0.001
				Treatment effect	F (2, 15) = 0.395	0.539
11j	8 mice - 9 mice	Two-way ANOVA (post-hoc Bonferroni)	Vehicle *vs* AM251	Interaction	F (1, 15) = 8.162	0.012
				Odor effect	F (1, 15) = 3.923	0.066
				Treatment effect	F (1, 15) = 1.632	0.221
				Odor 1'		>0.999
				Odor2		0.051

Supplementary References

1. Stark, E. *et al.* Inhibition-Induced Theta Resonance in Cortical Circuits. *Neuron* **80**, 1263–1276 (2013).

2. Stark, E. and Abeles, M. Unbiased estimation of precise temporal correlations between spike trains. *Journal of Neuroscience Methods* **179**, 90–100 (2009).

3. Girardeau, G., Inema, I. & Buzsáki, G. Reactivations of emotional memory in the hippocampus–amygdala system during sleep. *Nat Neurosci* **20**, 1634–1642 (2017).
