# Supplementary material for: Endogenous cannabinoids in the piriform cortex tune olfactory perception
Source: Nat Commun. 2024 Feb 9;15:1230. doi: 10.1038/s41467-024-45161-x (PMC10858223; doi:10.1038/s41467-024-45161-x)
Supplement: Supplementary file 3 — Inventory of Supplementary Information [file 41467_2024_45161_MOESM3_ESM.pdf]

Endogenous cannabinoids in the piriform cortex tune olfactory perception

Supplementary Information

Content:

- Supplementary Figures 1 to 11
- Supplementary Tables 1 and 2
- Supplementary References
